# Applying health belief model among mothers of children with thalassemia

**DOI:** 10.1038/s41598-025-15584-7

**Published:** 2025-08-25

**Authors:** Heba Said Mohamed Khawwas, Faten Khayrat El-Guindi, Nadia Hamed Farahat, Asmaa Talat Mohamed

**Affiliations:** Faculty of Nursing, Ain Shams University, Cairo, Egypt

**Keywords:** Children with thalassemia, Thalassemia, Mothers, Health belief model, Diseases, Health care

## Abstract

**Background:**

Thalassemia is a blood disorder that is transferred down through the generations. It has a negative impact on physical functioning of children and affect their social relationships and mental health. HBM has great potential in preventing thalassemia and its complication and promoting health level**.**

**Aim of the study:**

To evaluate the effect of health belief model among mothers of children with thalassemia.

**Design:**

A Quasi-experimental research design was utilized.

**Setting:**

The study was conducted at the outpatient haematology clinic at El-Menofia University Hospital.

**Subjects:**

A purposive sample of 106 mothers was used. Tools: Two tools were used to conduct this study 1^st^ Tool: interviewing questionnaire, 2^nd^ Tool: Health belief model scale.

**Results:**

53.8% of children had a family history of thalassemia, and there was improvement in total knowledge of mother’s knowledge as in preprogram was 28.4% and improved to 76.4% post program, and 72.6% in follow up. There were improvement in mother’s practices in caring of their children with thalassemia as in preprogram was 29.2% and improved to 80.2% post program, and 76.4% in follow up, and their health beliefs regarding thalassemia were improved as in preprogram was 17.0% and improved to 77.4% post program, and 73.6% in follow up.

**Conclusion:**

There was highly statistically significant with positive correlation between total knowledge and total practice pre, post and Follow up. Also, there was highly statistically significant with positive correlation between Total knowledge and Total health beliefs pre, post and Follow up and positive correlation between Total practice and Total health beliefs pre, post and Follow up, this indicates that the mother’s knowledge, practices, and beliefs regarding thalassemia were improved by the program.

**Recommendation:**

The study recommended that, provide support and education programs for parents to improve the care provided to children with thalassemia.

## Introduction

Thalassemia describes a group of inherited blood disorders associated with the alpha and beta globulins of hemoglobin. Alpha thalassemia and beta thalassemia result from the reduction of either alpha or beta globulins, respectively. Both types of thalassemia result in moderate to severe anemia and extra-medullary hematopoiesis, leading to signs and symptoms such as poor growth and development; skeletal deformities; thrombosis; pain in the head, back, and legs; impaired cardiac function; hepatosplenomegaly; non-transfusional iron overload; and conditions such as hydrops fetalis^1^.

Worldwide, every year around 70,000 infants are born with beta-thalassemia. About 270 million carriers of haemoglobinopathies exist globally. Beta-thalassemia is mostly prevalent among individuals of Mediterranean countries, as well as in Southeast Asia, India, Africa, Central America, and the Middle East. According to the World Health Organization (WHO) guidelines, published in 1998, genetic screening should not be compulsorily carried out. However, some countries including Iran,

Saudi Arabia, Palestinian Territories, and Cyprus now have mandatory premarital screening laws for hemoglobin disorders for all couples before marriage. Successful awareness programs have been carried out in countries like Greece, Italy, and Cyprus to reduce high carrier rates^2^.

One of the most-reported risk factors of thalassemia is consanguineous marriage. Consanguineous marriage is the marriage of two individuals having connections as second cousins. Patients with thalassemia minor show no manifestations and can have a healthy life without treatment, whereas patients with thalassemia major usually experience lifelong anemia that begins in early childhood, and due to the abnormality of the red blood cells, the patient must be managed with regular blood transfusions^3^.

Thalassemia is known as one of the blood disorder diseases that is inherited by parents. There are several types of Thalassemia, namely as Thalassemia major, minor, and inter-media. Among them, Thalassemia major is the most dangerous and needs more attention. Generally, it can be detected since the child is one year old. Late detection of this disease can have adverse consequences and various complications^4^.

Thalassemia has a negative impact on physical functioning of children and adolescents. It can also affect social relationships and mental health, eventually leading to poor school performance and overall impairment in the health related quality of life^5^.

The Health Belief Models (HBM) is a concept of individual perceptions which able to influence feedback behavior in decision making regarding their health conditions. The HBM theory according to Rosenstock consists of several types of perceptual models, including perceived susceptibility, severity, threats, benefits, barriers, and self-efficacy. The concept of HBM theory states that perceived susceptibility and severity of an illness can affect the perceived of threats^6^***.***

Nurses play a crucial role in improving the mothers‟ level of understanding about Thalassemia, through counseling program. Nurses have an important role in educating the mothers and focusing on their strength points and capacities, educational needs. Therefore, enhancing mothers knowledge regarding control of symptoms, minimizing drug side effects, participation in physical activities and living a normal life and improving their participation in the process of care to enhance quality of life and the abilities of mothers^7^.

Significance of the study.

Thalassemia is the most prevalent cause of chronic hemolytic anemia in Egypt, which has a high morbidity and mortality rate. Meanwhile, in Egypt, β -thalassemia major is a prevalent health problem; it is predicted that 1000 children with β- thalassemia are born per 1.5 million live births. In Egypt, carriers of β thalassemia account for 9–10 percent of the population. It is anticipated that 50,000 to 100,000 of children less than five years die yearly, accounting for 0.5–0.9 percent of all mortality in low- and middle-income nations^8^.

In Egypt, thalassemia is the most common genetically inherited hemoglobin disorder with a carrier rate ranging from 0.5% to more than 9% and 1000 children out of 1.5 million live births are born annually with thalassemia major^9^.

Aim of the study.

The study aims to evaluate the effect of health belief model among mothers of children with thalassemia through:

1. Assessing the mother’s knowledge regarding thalassemia.
2. Assessing the mother’s reported practices regarding care of their children with thalassemia.
3. Assessing the mother’s beliefs regarding thalassemia.
4. Design and implement health belief model based on previously detected knowledge and health beliefs of mothers of children with thalassemia.
5. Evaluating the effectiveness of health belief model on their improvement of knowledge, reported practices, and health beliefs of the mothers having children with thalassemia.

## Research hypotheses

Application of the health belief model among mothers having children suffering from thalassemia will improve their knowledge, improve their health beliefs and reported practices regarding their children with thalassemia, and prevent complication.

### Subjects and methods

#### The subjects and methods used for achieving the study was portrayed under four main designs as follows

1. Technical design.
2. Operational design.
3. Administrative design.
4. Statistical design.

##### Technical design

Includes research design, setting of the study, subjects of the study and tools for data collection.

##### Research design

A quasi- experimental study design was used to evaluate the effect of the application of health belief model for mothers of children with thalassemia regarding their knowledge, their health beliefs and their practices.

##### Setting

The current study conducted at the outpatient haematology clinic at El-Menofia University Hospital because it considered the largest hospital in El-Menofia governorate and also cover care service of all residents in Shbeen El Koom City and all villages around it.

##### Type of sample

A purposive sample was used within inclusive criteria.

##### Inclusion criteria

- Confirmed diagnostic children with thalassemia from both genders.
- Aged from 1to 12 years.
- Diagnosed from 6 months to 1year and accompanied by their mother.

##### Sample technique

The sample was collected randomly by simple random sample according to the previous criteria by using homogeneous sampling technique (involves selecting what is often a more narrow sample of individuals or units that are similar or have the same characteristics or attributes).

##### Sample size

106 mothers of children with thalassemia estimated according to sample size equation to achieve a confidence level of 95%. The total number of children with thalassemia attends to the previously mentioned setting and with the inclusion criteria in 2022 were 145 children (Ismail, 2022).

$$ n = \frac{{\text{N}}}{{1 + N(e)^{2} { }}} $$

n = Number of samples.

N = Total population.

e = Error tolerance (fixed number = 0.05)

$$ n = \frac{145}{{1 + 145(0.05)^{2} { }}} = {1}0{6}.{422 } \approx \,{1}0{\text{6 mother}} $$

###### Data collection tools

Two tools were used to conduct this study, it was designed after reading related and recent literature and taking expert’s and supervisors’ opinion, it was written in Arabic language.

**First tool:** A structured interviewing questionnaire. This tool contained four parts as follow:

**Part I:**

Socio-demographic characteristics of the children with thalassemia including age, gender, birth order, educational level.

Socio-demographic characteristics of mothers of children with thalassemia including age, educational level, occupational, marital status and number of family members.

**Part II:**

Medical history of the children with thalassemia: including family history & degree of consanguinity, duration of illness and family history of thalassemia.

**Part III:**

Mothers’ knowledge regarding thalassemia it was adapted from^10^ and was modified by the investigator to meet the aim of the study including definition, sign and symptoms, management, complications, prevention of disease, prevention of complications, knowledge about blood transfusion (Hb level which determine the need for blood transfusion, importance, complications, causes of complications, precaution to avoid complications), nutrition of child with thalassemia, and physical activity of child with thalassemia.

#### Scoring system:

Total knowledge questions included 22 questions. The mother’s knowledge checked with a model key answer and, accordingly, the mother’s knowledge categorized into either “correct answer was scored with a single point” or “incorrect answer and don’t know was scored with zero points”. These scores was summed and converted into a percentage score, classified into 2 categories: satisfactory knowledge if score ≥ 50%, unsatisfactory knowledge if score < 50%.

### Part IIV: Practices assessment

Mothers of children with thalassemia practices regarding care of their children with thalassemia: it divided into two parts reported practices and observed practices.

- **Reported practices**: It was adapted from Dathan et al.,^11^ and modified by the investigator to meet the aim of the study. It Included 8 questions about healthy diet, 9 questions about prevention of complications, 5 questions about management of fever, and 6 questions about hand washing and maintain oral care.
- **Observational chick list: it** was adapted from: Bowden and Greenberger,^12^, and modified by the investigator to suit the nature of study and reviewing from the researcher supervisors to assess observed practices of mothers regarding to care of their children with thalassemia. It included two procedures divided into,oral drug administration (9 steps), and mother’s follow up after blood transfusion (12 items).

### Scoring system:

Total practices questions included 51 questions. The done answer was scored (2 point), the not done answer was scored (1 point). The total answer was categorized into Adequate practices ≥ 60%, and inadequate practices < 60%.

**Second tool:** Health belief model scale: to assess the mother’s health beliefs regarding thalassemia adapted from^13^*,* and modified by the investigator to meet the aim of the study. Including:

- Perceived susceptibility of negative consequences of thalassemia (7 items).
- Perceived seriousness of thalassemia and its complications (9 items).
- Perceived benefits of the actions to recommended behaviours in managing thalassemia (11items).
- Perceived barriers to change one’s behaviours (6 items).
- Cues to action, indicating how respondents receive instruction about thalassemia (7 items).

### Scoring system:

Total scale items included 40 questions. Possible responses was measured using a three-point likert scale (agree = 2, neutral = 1, and disagree = 0) their health beliefs was categorized into good > 75%, average 60–75% and poor < 60%.

**2. Operational Design:**

Includes preparatory phase, content validity, pilot study and field work.

Preparatory phase:

Includes reviewing of recent, current, national and international related literature and theoretical knowledge of various aspects of the study using books, articles, scientific journal and internet with the aim of acquiring in-depth knowledge about the study.

Content validity:

Content validity done for the tools by 3 experts of professors in family and community health nursing department faculty of nursing Ain Shams university to test the tools for appropriateness, comprehensiveness, and applicability, and the necessary modifications was done accordingly.

Content reliability:

The previous tools were tested by Cronbach Alpha reliability analysis of the Used Tool.

**Table:** 

Items	Alpha Cronbach	F	P-value
Total knowledge score	0.845	22.127	< 0.001*
Total Practice score	0.792	28.210	< 0.001*
Total health beliefs score	0.813	25.471	< 0.001*

This table show reliability in knowledge, practice and health beliefs when Cronbach alpha was > 0.5.

### Tools reliability

The reliability was scaled as follows: < 0–0.25 weak reliability, 0.25–0.75 moderate reliability, 0.75- < 1strong reliability and 1 is optimum. The reliability for this questionnaire was 0.82.

#### Pilot study

It was carried out on 10%, 11 of mothers of children with thalassemia to test applicability, feasibility, and time needed to collect data of the tools, mothers in the pilot study chosen randomly and then was included in the study sample as, There were no modifications found after pilot study. The pilot showed very high levels of reliability. The final forms of tools were obtained and time needed for completing the tools was determined about 30–40 min.

#### Ethical consideration.

Permission for data collection and implementation of the instructional guidelines was obtained from scientific ethical committee in faculty of nursing at Ain Shams University before starting the study and gave its approval to this study (code number 24.03.238) based on the standards of the committee and adhered to the Declaration of Helsinki. The Dean of the Faculty of Nursing/Ain Shams University sent official letters to the medical and nursing managers of El-Menofia University hospitals in El-Menofia city/Egypt, requesting their approval and collaboration for executing the study and gathering the data. These letters clarified the study’s purpose and its procedures. Informed written consent was received from each mother intern after explaining all the study phases and being instructed about her right to leave the study without giving rationales. The mothers interns were guaranteed the anonymity and confidentiality of the data gathered.

#### Program Construction

The present study was conducted in four phases:

First: Preparatory phase:

Review of recent, current, national and international related literature in various aspects of thalassemia in this phase to design the study tools and booklet.

Second: Assessment phase:

The assessment done to determine the mothers of children with thalassemia needs by using pretest based on the collecting data on the mother’s knowledge, behaviors and their reported and observed practice regard caring of their children with thalassemia.

#### Planning and implementation phase

In this phase, planning and implementation of the program and its content according to its objectives, addressing the knowledge and practices through using the booklet to give the information and using different methods as Lectures, demonstration, re-demonstration, role play & group discussion to illustrate and explain the knowledge and practice that are necessary for increasing the mothers awareness toward the caring of their children with thalassemia through the sessions that was given twice weekly within 8 sessions, each session took from 30-60min.

#### The program objective

Increase the mother’s knowledge, practices and, improve their health beliefs regarding thalassemia.

#### Program content

The program content help the mothers of the children with thalassemia to gain the basic knowledge, applying proper practices related to the health needs of their children and improve their health believes.

#### Theories sessions

The educational program structure and its objectives to improve the mothers knowledge about thalassemia: its definition, causes, risk factors, signs and symptoms, diagnosis, complications and treatment. Also improve the mother’s knowledge about blood transfusion: its definition, importance, complications, and the precautions during blood transfusion, determine the component of healthy food for thalassemia, importance of exercise for children with thalassemia.

#### Practices sessions

This part of the program was aimed to improve mother’s practices regarding care of their children with thalassemia it was included: apply proper hand washing, apply measuring of axillary temperature, display correct way of drugs administration and application of health belief model for children with thalassemia.

### Program implementation

The researcher visited the selected setting from9.00 am to 1.00pm two days/ week for program implementation. The program content and its objective were developed by the researcher in form of 8 sessions each session take about 30–45 Mins according to the mothers understanding and attention span. Teaching sessions were conducted in the clinic and waiting area.

At the beginning of the first session, an orientation about the program and its purpose was given. It was agreed at the time of the sessions with the mothers, from the second session and so on each session started by a summary about what was given through the previous session and objective of new one. By the end of each session a summary was made.

Each session of the program contained of general and specific objective, these objectives achieved through several teaching methods and media as lecture, group discussion, brain storming, posters, guidance booklet, illustrative pictures and hand out which includes instruction and information for mothers as a reference during and after program implementation.

Since it was difficult to fit all the study subjects at the same time in the program, this program implemented through 8 sessions. All study sample divided to 6 groups, each group contained from 17 to 18 mother. The investigator discussed the all session through two days weekly for along 8 weeks.

Fourth: Evaluation Phase:

This phase aims to evaluate the effect of health belief model among mothers of children with thalassemia. A post–test, was the same as pre-test and was given to the mothers after accomplishment of the training program through one month period of time. Then the investigator made the follow up after three month.

### Administrative design

An official approval letter was taken from the Dean of faculty of Nursing –Ain Shams University to the general manager of the previously mentioned setting to facilitate the conduction the study.

### Field Work

The researcher met the general manager of El-Menofia University Hospital at El-Menofia governorate to explain the aim of the study to facilitate the researcher`s work. After permission of the manager, the researcher started with introducing herself and explaining the aim of the study for the selected mothers of children with thalassemia, assured the data collected will be confidential and would be used only to achieve the purpose of the study. The field work was carried out within duration 6 months starting from the September 2023 till the end of February 2024. The researcher visited the pre-mentioned setting two days per week “Monday and Wednesday” for collecting the data from (106 mothers), it lasted one month to be fulfilled before implementation of the instructional guidelines. The implementation phase of the program phase of the instructional guidelines lasted for one month through visited the pre-mentioned setting two days every week (Monday and Wednesday) to accomplished and teaching sessions were conducted at the clinic and waiting area. The evaluation phase of the instructional guidelines lasted immediately for one months to determine the level of improvement for mother`s awareness regarding thalassemia then the researcher made the follow up after three months.

### Statistical design

Statistical presentation and analysis of the present study was conducted, using the mean, standard deviation, unpaired student t-test was used to compare between two groups in quantitative data, chi-square test was used to compare between groups in qualitative data, ANOVA test was used for comparison among different times in the same group in quantitative data, linear correlation coefficient was used for detection of correlation between two quantitative variables in one group. By (*IBM SPSS Statistics for Windows, Version 20.0. Armonk, NY: IBM Corp.)*.

Significant level: > 0.05 Non significant < 0.05* significant < 0.001* High significant.

## Results

### Part I: socio-demographic characteristics

Table 1 Reveals that, 51.0% of children with thalassemia their age ranged between 6–12 years with Mean ± SD = 4.67 ± 1.5. Also 58.5% of children were male, followed by 51.0% of them were at primary school.

**Table 1:** Distribution of studied children with thalassemia according to their socio demographic characteristics (n = 106).

Socio demographic characteristics		N	%
Age	1 ≤ 3 years	19	17.9
	3 ≤ 6 years	33	31.1
	6–12 years	54	**51.0**
	Mean ± SD	4.67 ± 1.5	
Gender	Male	62	**58.5**
	Female	44	41.5
The child’s order	the first	50	47.2
	the second	22	20.8
	the third	25	23.6
	the fourth or more	9	8.5
Educational level for the child	Before nursery age	26	24.5
	Nursery age	26	24.5
	primary school	54	**51.0**

Table 2 Reveals that, 50.9% of mothers their age ranged between 25 ≤ 40 years with Mean ± SD = 32.42 ± 4.65. Also 54.7% of women were Intermediate education, followed by 69.8% of them were married, and 63.2% were not working. The same table shows that, 67.9% of them with not enough monthly income.

**Table 2:** Distribution of studied mothers according to their socio demographic characteristics (n = 106).

Socio demographic characteristics		N	%
Age	18 ≤ 25 years	22	20.8
	25 ≤ 40 years	54	**50.9**
	More than 40 years	30	28.3
	Mean ± SD	32.42 ± 4.65	
Educational level	Uneducated	25	23.6
	Basic education	18	17.0
	Secondary education	58	**54.7**
	University education/or more	5	4.7
Marital status	Married	74	**69.8**
	Divorced	12	11.3
	Widow	20	18.9
Occupation	Working	39	36.8
	do not work	67	**63.2**
Income	Enough	34	32.1
	Not enough	72	**67.9**
Number of family members	From 3 and less than 5 individuals	50	47.2
	From 5–7 individuals	56	**52.8**

### Part II: Medical history of the child related to thalassemia

Table 3 Presents that, 46.2% of the studied children didn’t have consanguinity between their parents. While 53.8% of children had a family history of thalassemia. In addition, 53.8% of the children the disease was discovered from symptoms of the disease.

**Table 3:** Distribution of studied sample of children with thalassemia according to their family and past medical history. Reported by mother. (n = 106).

Family history		N	%
Degree of consanguinity between parents	The first	41	38.7
	Second	16	15.1
	There is no degree of consanguinity	49	**46.2**
Presence of family history of thalassemia	Yes	57	**53.8**
	No	49	46.2
Past child medical history			
In case of presence of family member affected (n = 57) (Results not mutually exclusive)	the father	35	**61.4**
	the mother	22	38.6
	Brother	11	19.3
	Uncle	12	21.1
Discovering the disease	Accidentally	39	36.8
	Symptoms of the disease	57	**53.8**
	Complications of the disease	10	9.4
Removing of the spleen	Yes	39	**36.8**
	No	67	63.2
The child’s age at the time of the spleen-ectomy. (n = 39)	≤ Three years	10	25.6
	> Three years	29	**74.4**

Also the table illustrates that, 36.8% of the children had spleen-ectomy and 74.4% of them had remove the spleen when they were more than three years.

Table 4 Shows that, 38.7% of the children their BMI levels 30–41.8 with Mean ± SD = 24.3 ± 3.95. While this table prove that, 73.6% of the children had blood transfusion, and 35.8% of children transfuse blood regularly. The same table reflects that, 41.5% of the children checked the blood iron level as doctor order.

**Table 4:** Distribution of studied sample of children with thalassemia according to their current medical history. Reported by mother and from the medical records (n = 106).

Current medical history		N	%
BMI	Mean ± SD	24.3 ± 3.95	
The child having blood transfused	Yes	78	**73.6**
	No	28	26.4
If the answer is yes, the blood transfusion routine. (n = 78)	Regularly (per three weeks or per month)	42	**53.8**
	when child is needed as doctor order	19	24.4
	When the child become very sick	17	21.8
Time of checking blood iron level	Every 3 months	37	34.9
	Every 6 months	25	23.6
	as doctor order	44	**41.5**
current treatment	Medicines	28	26.4
	blood transfusion and medication	78	**73.6**
Taking medications to get rid of iron accumulation in the body	Yes	65	**61.3**
	No	41	38.7
The child taking folic acid and vitamins B continuously	Regular	94	**88.7**
	Irregular	12	11.3
Current complications resulting from thalassemia (Results not mutually exclusive)	Enlarged spleen	19	17.9
	Slow growth rates	20	18.9
	Spleen-ectomy	39	36.8
	Iron accumulation in the blood	32	**30.2**
	There is not any complications	36	33.96
Commitment of follow up	Always	59	**55.7**
	Sometimes	37	34.9
	Never	10	9.4

Also the table illustrates that, 73.6% of the children their current treatment was blood transfusion and medication. 66.0% of the children were taking medications to get rid of iron accumulation in the body, while 88.7% were taking folic acid and vitamins B regularly. Finally, there were 55.7% of the children always follow up regularly.

### Part III: Mothers’ knowledge regarding thalassemia

**Fig. 1:**
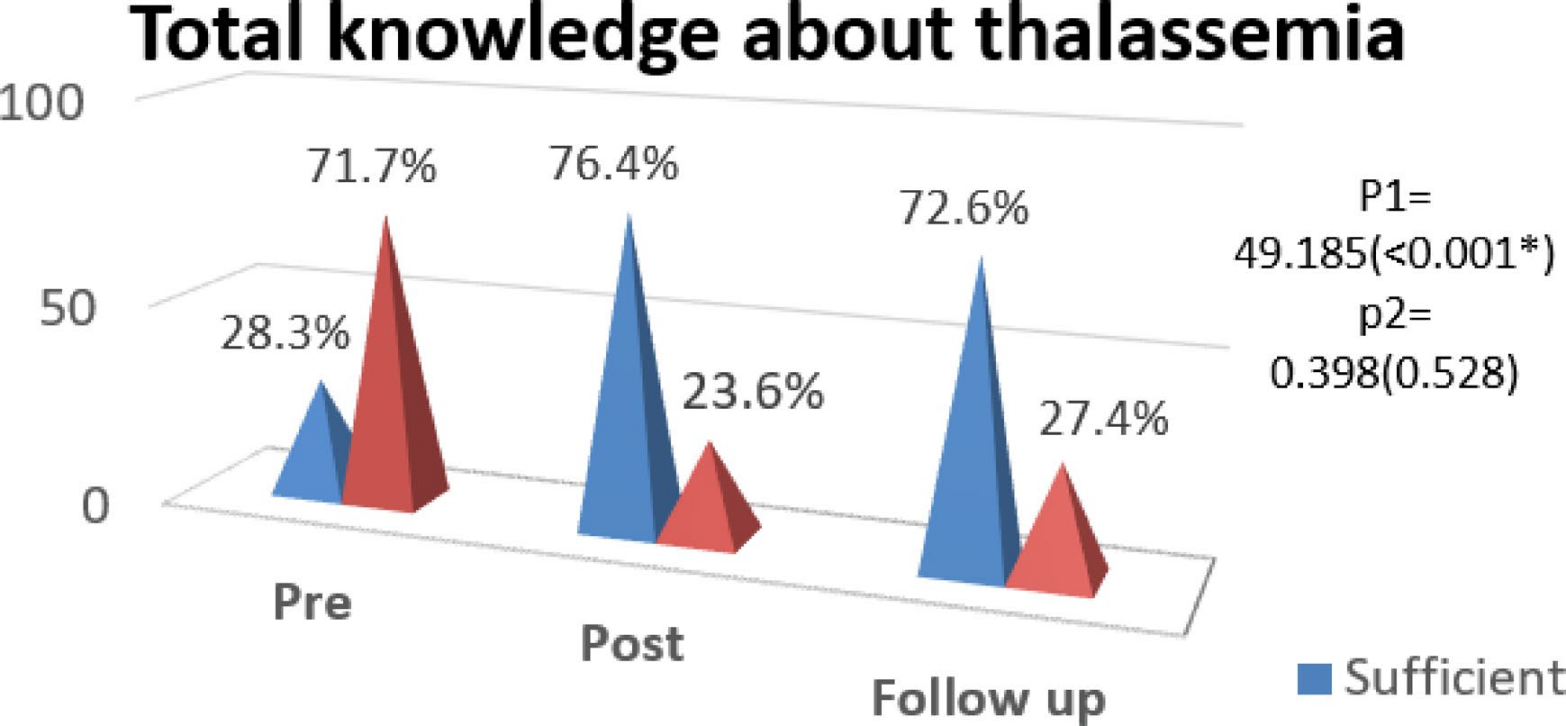
Distribution of studied sample of mothers of children with thalassemia according to their total knowledge. (n = 106).

Figure 1 proves that, 76.4% of the participated mothers had satisfactory total knowledge about thalassemia post program compared to 28.3% of them pre the program.

Also the figure illustrates that, there was a statistically significant difference between total knowledge about thalassemia through pre-program and post program implementation phases with p < 0.001*, and there was no statistically significant difference between total knowledge about thalassemia through post program implementation and follow up phases with p value = 0.528.

### Part IV: Mother’s reported practices regarding care of their thalassemic children

**Fig. 2:**
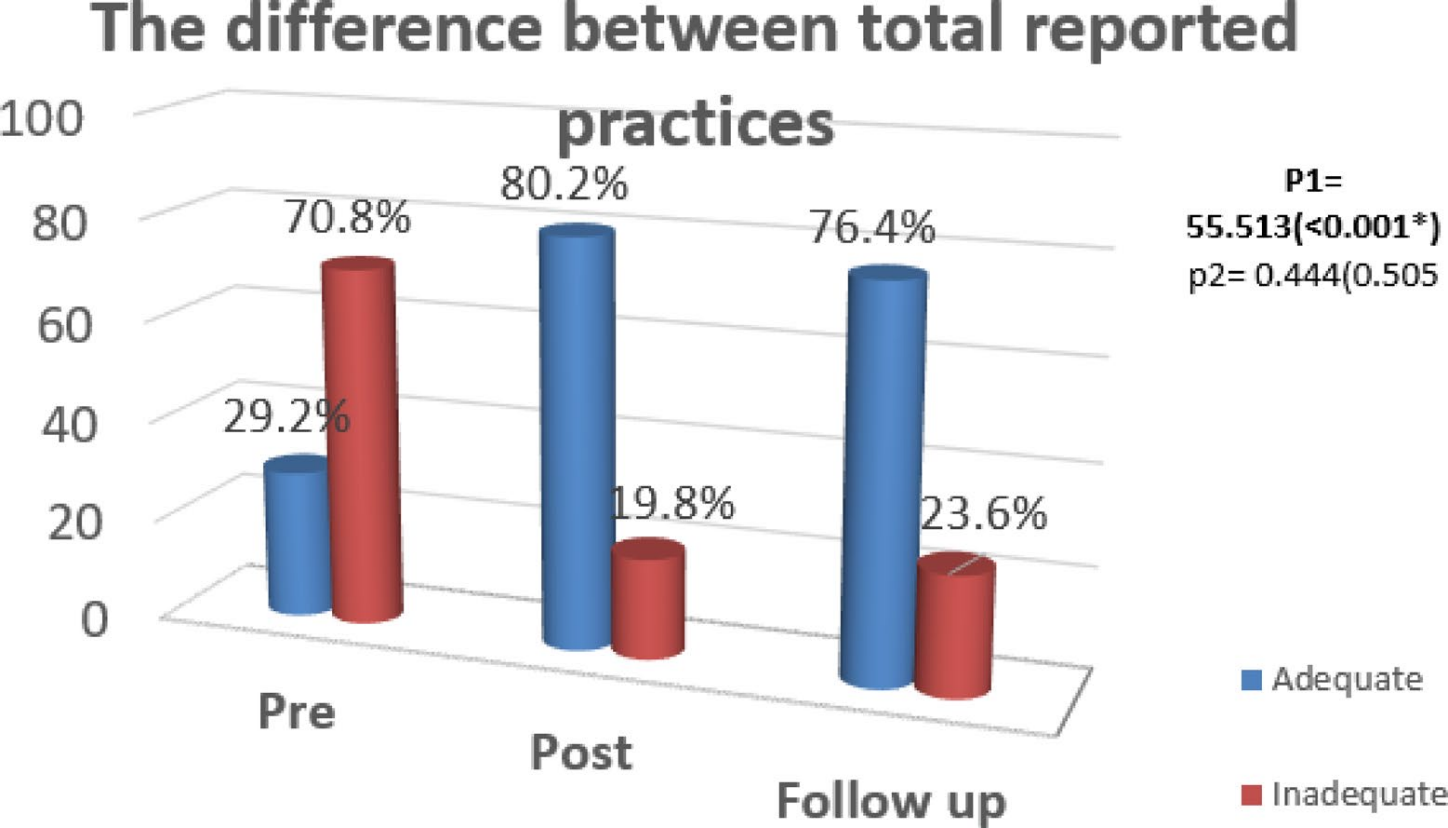
Difference between total reported practices levels of mothers of children with thalassemia during implementation phases (n = 106).

Figure 2 proves that there was a highly statistically significant difference between total practices about caring of the children with thalassemia through pre-program and post program implementation phases with p < 0.001*, and there was no statistically significant difference between total practices through post program implementation and follow up phases with p value = 0.505.

As well as the figure shows that, greatest 80.2% of the mothers participated in the study had adequate practices post program compared to 29.2% of them before program.

### Part V: Health belief model of the mothers regarding thalassemia

**Fig. 3:**
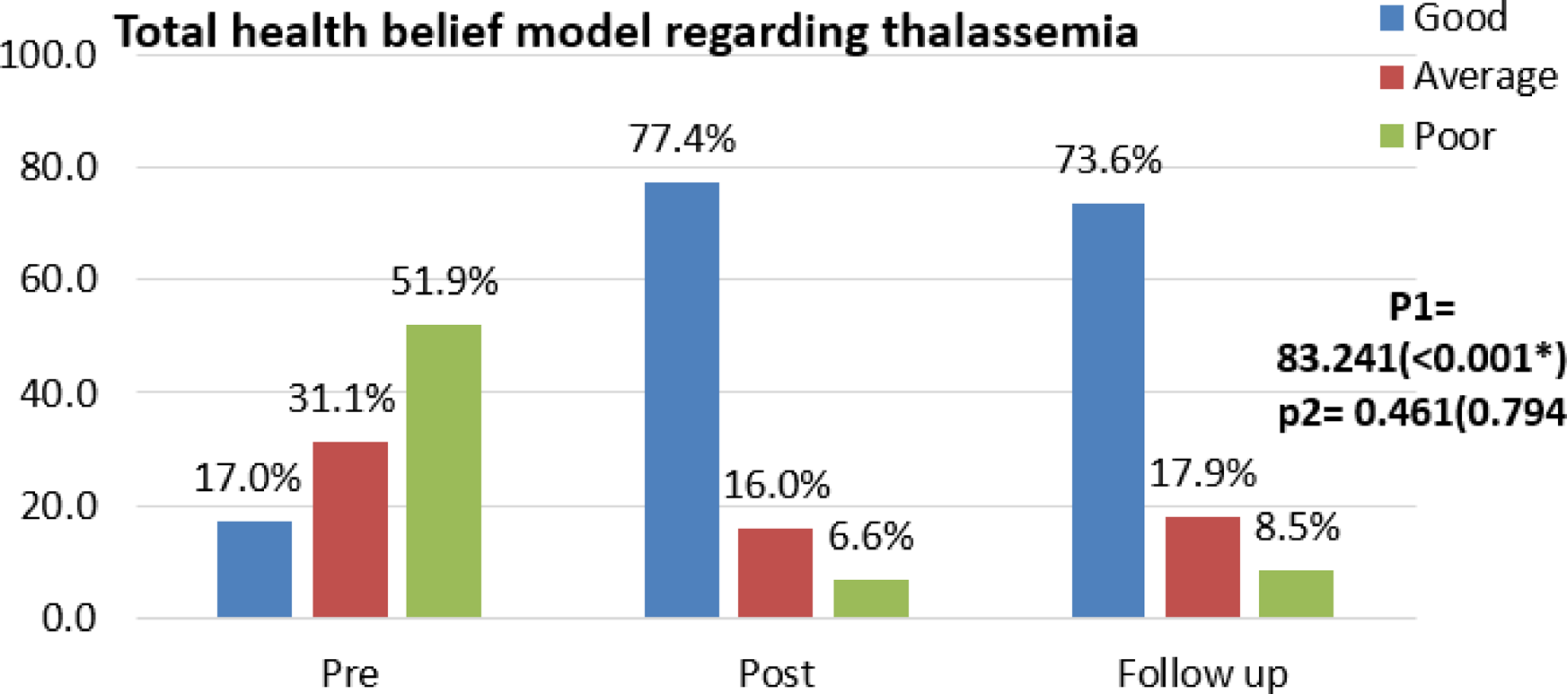
Difference between total health beliefs of mothers of children with thalassemia during program implementation phases (n = 106).

Figure 3 prove that there was a highly statistically significant difference between total health beliefs regarding thalassemia through pre-program and post- program implementation phases with p < 0.001*.

The figure also illustrated that, more than three quarters 77.4% of the mothers participated in the study had good health beliefs post program compared to 17.0% of them pre-program.

### Part VI: Statistical relation between study variables

Table 5 Proves the effectiveness of program implementation through the noticed a statistically significant relationship between mother’s knowledge level during pre, post, and follow up of the program and their total practice with p ˂0.001*.

**Table 5:** The relation between total knowledge of mothers of children with thalassemia, and their total practice in pre, post and during follow up program implementation phases (n = 106).

Total practice	Total knowledge					
	**Sufficient**		**Insufficient**		**Chi-square**	
	**N**	**%**	**N**	**%**	**X**^**2**^	**P-value**
**Pre**						
Adequate	27	25.5	4	3.8	74.638	< 0.001*
Inadequate	3	2.8	72	67.9		
**Post**						
Adequate	81	76.4	4	3.8	84.850	< 0.001*
Inadequate	0	0.0	21	19.8		
**Follow up**						
Adequate	74	69.8	7	6.6	60.537	< 0.001*
Inadequate	3	2.8	22	20.8		

*Significant relation.

Table 6 Proves the effectiveness of program implementation through the noticed a statistically significant relationship between mother’s knowledge level during pre, post, and follow up of the program and their total health beliefs with p ˂0.001*.

**Table 6:** The relation between total knowledge of mothers of children with thalassemia, and their total health belief in pre, post and during follow up program implementation phases (n = 106).

Total health beliefs	Total knowledge					
	Sufficient		Insufficient		Chi-square	
	N	%	N	%	X^**2**^	P-value
Pre						
Good	18	17.0	0	0.0	68.367	< 0.001*
Average	12	11.3	21	19.8		
Poor	0	0.0	55	51.9		
Post						
Good	78	73.6	4	3.8	71.180	< 0.001*
Average	3	2.8	14	13.2		
Poor	0	0.0	7	6.6		
Follow up						
Good	72	67.9	6	5.7	59.593	< 0.001*
Average	5	4.7	14	13.2		
Poor	0	0.0	9	8.5		

*Significant relation.

Table 7 Proves the effectiveness of program implementation through the noticed a statistically significant relationship between mother’s practice level during pre, post, and follow up of the program and their total health beliefs with p ˂ 0.001*.

**Table 7:** The relation between total practice of mothers of children with thalassemia, and their total health belief in pre, post and during follow up program implementation phases (n = 106).

Total health beliefs	Total practice					
	Adequate		Inadequate		Chi-square	
	N	%	N	%	X^2^	P-value
**Pre**						
Good	18	17.0	0	0.0	67.924	< 0.001*
Average	13	12.3	20	18.9		
Poor	0	0.0	55	51.9		
**Post**						
Good	82	77.4	0	0.0	90.448	< 0.001*
Average	3	2.8	14	13.2		
Poor	0	0.0	7	6.6		
**Follow up**						
Good	75	70.8	3	2.8	67.216	< 0.001*
Average	6	5.7	13	12.3		
Poor	0	0.0	9	8.5		

*Significant relation.

Table 8 There was highly statistically significant with positive correlation between Total knowledge and Total practice pre, post and Follow up (r = 0.785 P < 0.001*, r = 0.622 P < 0.001*, r = 0.485 P < 0.001*) respectively.

**Table 8:** Correlation between knowledge, practice and health beliefs scores at pre, post and Follow up (n = 106).

	**Total knowledge**		**Total practice**	
	**r**	**P-value**	**R**	**P-value**
**Pre**				
Total practice	0.785	< 0.001*		
Total health beliefs	0.725	< 0.001*	0.537	< 0.001*
**Post**				
Total practice	0.622	< 0.001*		
Total health beliefs	0.562	< 0.001*	0.660	< 0.001*
**Follow up**				
Total practice	0.485	< 0.001*		
Total health beliefs	0.354	< 0.001*	0.588	< 0.001*

Also there was highly statistically significant with positive correlation between Total knowledge and Total health beliefs pre, post and Follow up (r = 0.725 P < 0.001*, r = 0.562 P < 0.001*, r = 0.354 P < 0.001*) respectively.

At the same tale there was highly statistically significant with positive correlation between Total practice and Total health beliefs pre, post and Follow up (r = 0.537 P < 0.001*, r = 0. 660 P < 0.001*, r = 0.588 P < 0.001*) respectively.

## Discussion

In Egypt, beta thalassemia major is considered the most common chronic hemolytic-anemia with an estimated carrier rate of 9–10.2% of the population. The disease represents a significant physical, psychological and financial burden on the affected families and the Egyptian government. It also requires significant resources and is considered a challenge in constrained healthcare systems^14^*.*

### Medical history of the children related to thalassemia & their families

With regards to the degree of consanguinity between parents, the result of the current study illustrated that nearly to half of the studied sample didn’t have consanguinity between their parents (Table 3). This finding was reinforced by Silva & Peiris,^15^, who assessedv the “health-related quality of life of children with thalassemia major in Two Selected Hospitals in in Sri Lanka” applied on 60 children with thalassemia, were revealed that, the Consanguinity was negative in 86.7% of the parents.

Related to presence of family history of thalassemia, the result of the present study revealed that more than half of the studied children had family history of thalassemia (Table 3). This findings were in same line with study by Mahmoud et al.,^16^ who conducted a study on 120 participants about “Detection of endocrine disorders in young children with multi-transfused thalassemia major, in Egypt “were reported that the prevalence of presence of family history was 61.7% among the studied children. Also, this finding disagreed with study by Mat et al.,^17^ entitled “Parental knowledge on thalassaemia and factors associated with refusal to screen their children, in Malaysia” applied on 273 parents about were reported that the prevalence of presence of family history was 5.1% among the studied children.

Regarding to removing of the spleen, the result of present study illustrated that more than one third of children had removed of the spleen (Table 3). This finding is disagree with study by Mahmoud et al.,^16^ who conducted a study on 120 participants entitled “Detection of endocrine disorders in young children with multi-transfused thalassemia major, in Egypt”, were reported that near to half (45.8%) of the studied children had splenectomy.

As regard to The child’s current medical history, regarding to the child having blood transfused, the result of the current study illustrated that nearly to three quarters of the studied children had blood transfusion (Table 4). This result supported by Hassan et al.,^18^ who conducted a study on 23 children which entitled “Study of the health instructions effect on quality of life and psychological problems among children with thalassemia “, were reported that the most of the studied children (82.6%) had blood transfusion.

Related to take medications to get rid of iron accumulation in the body, the result of this study revealed that more than half of the studied sample were taking medications to get rid of iron accumulation in the body (Table 4)^19^. This finding in same line with study by Shafie et al.,^20^ who conducted a study which entitled about “Health-related quality of life among children with transfusion-dependent thalassemia: a cross-sectional study in Malaysia. “ applied on 368 children, were reported that more than two thirds (72.8%) of the studied children had medications to get rid of iron accumulation in the body.

Regarding to the child taking folic acid and vitamins B continuously, the result of present study illustrated that the most of children had taking folic acid and vitamins B regularly. (Table 4). This finding in same line with the study by Al-Hakeim et al.,^21^ who conducted a study on 111 children about “Major depression in children with transfusion-dependent thalassemia is strongly associated with the combined effects of blood transfusion rate, iron overload, and increased pro-inflammatory cytokines, in Iraq”, were showed that the most of the children (79.1%) had taking folic acid and vitamins B continuously.

### Mother’s knowledge regarding thalassemia

Related to total mother’s knowledge regarding thalassemia, the result of present study showed that, more than three quarter of the participated mothers had satisfactory total knowledge about thalassemia post program compared to more than one quarter of them pre-program with a statistically significant difference (Fig. 1). This finding supported with study by Mohammed & Abdalla,^8^ which entitled “effect of health coaching intervention on mothers’ performance and quality of life of their children with beta thalassemia, Egypt” applied on 70 mothers of children with thalassemia, were revealed that the greatest percentage (82.9%) of the participated mothers had satisfactory total knowledge about thalassemia after health coaching intervention compared to 32.9% of them before.

From researcher`s point of view, The consistently high level of significance across all items of knowledge emphasizes the effectiveness and enduring impact of the educational program. These findings underscore the success of the intervention in enhancing participants’ understanding of various aspects related to thalassemia, contributing to a substantial increase in knowledge levels that persisted even at the follow-up assessment.

### Mother’s practices regarding caring of their children with thalassemia

In relation to the mother’s total practice in caring for their children with thalassemia, the current study’s findings revealed that, greatest of the mothers participated in the study had adequate practices post program compared to less than one third of them before program with a statistically significant difference Fig. 2. This result was in congruent with Grabmann & Schermuly,^22^ who reported in their study which entitled “A literature review on negative impacts of coaching what we know and what we need to know, in German” that participants who received occupational performance coaching improved their performance and satisfaction with their goals pre and post coaching. Also, The findings were consistent with those obtained by Kish et al.,^23^*,* who studied on 69 parents about “working and caring for a child with chronic illness: A review of current literature,Child: Care, Health and Development.” according to a study, the participants’ performance means scores were significantly different from the control group’s performance. According to the researchers’ point of view, this progression in mothers’ practice may be due to their great desire to learn more about illness in order to help their children care for them and improve their children’s quality of life.

### Health belief model of the mothers regarding thalassemia

In relation to the mothers’ total health beliefs regarding thalassemia, the current study’s findings revealed that, more than three quarters of the mothers participated in the study had good health beliefs post program compared to few of them pre-program with a statistically significant difference between total health beliefs regarding thalassemia through program implementation phases with p < 0.001*. (Fig. 3). This result was in congruent with Kia et al.,^24^ who conducted a study on 224 cases about “Evaluation of an educational intervention based on health belief model on beta thalassemia carrier and final suspects couples in Andimeshk City “, were reported that participants who received educational intervention program improved their health beliefs with their goals pre and post program. According to the researchers’ point of view, this progression in mothers’ beliefs may be due to their great desire to learn more about illness in order to help their children care for them.

### Statistical relation between study variables

Regarding to relation between total knowledge of mothers of children with thalassemia, and their total practice, the present study revealed that there were noticed a statistically significant relationship between mother’s knowledge level during pre, post, and follow up of the program and their total practice with p ˂0.001* (Table 5). This results supported with study on 70 parents by Atshan & Aziz^25^ who conducted a study about “Impact of an educational program on parents’ knowledge about chelation therapy & nutrition of their children with beta thalassemia major. In Iraq” were reported that there high significant relation between mother’s knowledge level and their total practice during the program phases with p ˂0.001.

As regarding to relation between total knowledge of mothers of children with thalassemia, and their total health belief, the present study revealed that there were a statistically significant relation between mother’s knowledge level during pre, post, and follow up of the program and their total health beliefs with p ˂0.001* (Table 6). This finding supported with study by Masoudi et al.,^26^ who conducted a study on 100 participants about “The effect of health belief model-based training on preventing major thalassemia in thalassemia carrier couples. In Iran” were reported that there high significant relation between mother’s knowledge level and their total practice during the program phases with (P < 0.05).

Concerning to the relation between total practice of mothers of children with thalassemia, and their total health belief, the results of the present study revealed that there were noticed a statistically significant relationship between mother’s practice level during pre, post, and follow up of the program and their total health beliefs with p ˂0.001* (Table 7). This results agreed with study by Alanazi.,^27^ who conducted a study on 146 participants about “ The Health Belief Model to Predict Use of Hemoglobinopathies Preventative Behaviors, Among Premarital Saudis at risk for delivering A Child with Hemoglobinopathies”, were reported that there positive relationship between health beliefs and intention to adopt mother’s health practices. High significant association between socio- demographic characteristics of mothers and the overall items of health beliefs with P < 001.

Regarding to correlation between knowledge, practice and health beliefs scores, the present study revealed that there were there was highly statistically significant with positive correlation between total knowledge and total practice pre, post and Follow up (r = 0.785 P < 0.001*, r = 0.622 P < 0.001*, r = 0.485 P < 0.001*) respectively. (Table 8). This results was in line with study by Ezaat et al.,^28^ who conducted a study on 60 mothers about “Effect of Educational Program on Mothers’ Knowledge and Practices regarding Their Children with Splenomegaly. In Egypt” were reported that there significant positive correlation (p = 0.006) existed between total knowledge scores and total practice scores at all three time points, highlighting the program’s effectiveness in translating knowledge into improved care practices.

Regarding to correlation between knowledge, practice and health beliefs scores, the present study revealed that there were there was highly statistically significant with positive correlation between total knowledge and total health beliefs pre, post and follow up (r = 0.725 P < 0.001*, r = 0.562 P < 0.001*, r = 0.354 P < 0.001*) respectively (Table 8). This results supported with study by Shaimaa et al.,^29^ who conducted a study on 340 participants about “Effect of Application of Health Belief Model on females’ Knowledge and Practice regarding the premarital counselling Regarding Thalassemia. In Egypt” were reported that there was a positive highly statistically significant correlation between total knowledge and total health beliefs scores in both intervention and control groups before and after two months of application implementation p 0.000.

## Conclusion

### On the light of the findings of the present study and research hypotheses, it can be concluded that

There was highly statistically significant with positive correlation between Total knowledge and Total practice pre, post and Follow up (r = 0.785 P < 0.001*, r = 0.622 P < 0.001*, r = 0.485 P < 0.001*) respectively, there was highly statistically significant with positive correlation between total knowledge and total health beliefs pre, post and follow up (r = 0.725 P < 0.001*, r = 0.562 P < 0.001*, r = 0.354 P < 0.001*) respectively. Also there was highly statistically significant with positive correlation between total practice and total health beliefs pre, post and follow up (r = 0.537 P < 0.001*, r = 0. 660 P < 0.001*, r = 0.588 P < 0.001*) respectively.

## Recommendation

### Based on the findings of the current study, the following recommendations can be suggested

1. Increase awareness of mothers about preventive measures of thalassemia through premarital screening for discovering thalassemia carrier, genetic analysis, preventing consanguineous marriage, and prenatal screening to reduce the incidence of thalassemia by providing health education programs.
2. Providing educational guidelines, posters, pamphlets and manuals for mothers about thalassemia and encourage them to get use from them.
3. Further studies should be conducted with large sample in different settings to generalize the results.

## Data Availability

Due to confidentiality concerns, public access to the materials and data utilized in this study is not permitted. On reasonable request, they can be obtained from the corresponding author.

## Abbreviations

EKB: Egyptian knowledge bank
Hb: Hemoglobin
HBM: Health belief model
n: Number
REC: Research Ethics Committee
WHO: World Health Organization
